# A systematic review and meta-analysis of the use of oral zinc in the treatment of hepatic encephalopathy

**DOI:** 10.1186/1475-2891-12-74

**Published:** 2013-06-06

**Authors:** Norberto C Chavez-Tapia, Asunción Cesar-Arce, Tonatiuh Barrientos-Gutiérrez, Francisco A Villegas-López, Nahum Méndez-Sanchez, Misael Uribe

**Affiliations:** 1 Obesity and Digestive Diseases Unit, Medica Sur Clinic & Foundation, Mexico City, Mexico; 2 National Institute of Public Health, Mexico City, Mexico; 3 Center for Integrative Approaches to Health Disparities, University of Michigan, Ann Arbor, USA

**Keywords:** Therapy, Liver cirrhosis, Evidence-based medicine

## Abstract

**Background and aim:**

Because low serum zinc levels precipitate hepatic encephalopathy, zinc supplementation is considered a potential therapeutic option. The aim of this study was to assess the effects of oral zinc supplementation in the treatment of hepatic encephalopathy.

**Methods:**

For this systematic review and meta-analysis, data sources included electronic databases (CENTRAL, MEDLINE, EMBASE) and manual searching. Randomized clinical trials of adult patients diagnosed with liver cirrhosis and hepatic encephalopathy were included. The types of interventions considered were any oral zinc supplementation versus no intervention, placebo, or other interventions for the management of hepatic encephalopathy. The data were analyzed by calculating the RR for each trial and expressing the uncertainty as 95% CI. Continuous data were analyzed by calculating the standard mean differences (SMD) between groups within each trial and their 95% CI. Statistical heterogeneity was defined as a *P*-value > 0.10 (*χ*^2^) or *I*^2^ > 25%.

**Results:**

Four trials with a total of 233 patients were included. Oral zinc supplementation was associated with a significant improvement in performance on the number connection test (SMD –0.62; 95% CI –1.12 to –0.11) reported in three trials (n = 189), but not with encephalopathy recurrence reduction (RR 0.64; 95% CI 0.26 to 1.59) reported in two trials (n = 169). Other clinically significant outcomes (mortality, liver related morbidity, quality of life) were not reported.

**Conclusion:**

Oral zinc supplementation improved performance on the number connection test, but no evidence about other clinical or biochemical outcomes was available.

## Introduction

Hepatic encephalopathy is a neuropsychiatric complication of liver disease that affects 20 to 30% of the patients with cirrhosis [1,2], reducing health-related quality of life and causing a reversible decline in cognitive function. Previous studies have demonstrated that a reduction in blood ammonia levels improves hepatic encephalopathy, neuropsychological test performance, cognitive function, and health-related quality of life [3]. Lactulose, an ammonia absorption minimizer, has been successfully used to reduce blood ammonia levels in minimal hepatic encephalopathy. However, lactulose has no ammonia detoxification effect, rendering it ineffective to treat advanced hepatic encephalopathy [4-6].

Two major organs are involved in the metabolism of ammonia: the liver, in which ammonia is converted to urea via ornithine transcarbamylase, and the skeletal muscle, where ammonia is metabolized to glutamic acid via glutamine synthetase [5]. Zinc is a critical cofactor in these enzymatic reactions. Animal models have shown zinc deficiency decreases the activity of ornithine transcarbamylase, while zinc supplementation markedly increases hepatic ornithine transcarbamylase activity. Zinc deficiency has also been reported to impair the activity of muscle glutamine synthetase, which leads to hyperammonemia [6-8].

Zinc deficiency is observed frequently in patients with cirrhosis and hepatic encephalopathy [9]. Poor nutritional intake caused by a protein-restricted diet, impaired intestinal absorption, and excessive urinary loss are all potential causes of a low serum zinc levels in patients with advanced cirrhosis [5]. Short-term oral zinc supplementation may improve hepatic encephalopathy by correcting the zinc deficiency that compromises the conversion of ammonia to urea [10]. Bresci et al. reported better psychometric test performance in a zinc-supplemented group than in a standard therapy group, although the difference was not significant [11]. Similarly, oral zinc supplementation can improve hepatic encephalopathy in patients failing to respond to protein restriction and lactulose [2,6-8].

Zinc supplementation, in addition to standard therapies, may increase the hepatic conversion of amino acids into urea, decrease serum ammonia level, and consequently improve health-related quality of life. The effect of long-term oral zinc supplementation in addition to standard therapy on recurrent hepatic encephalopathy has not been established [7,8,12]. Despite the low cost and infrequent side effects of zinc supplementation, there is little evidence-based information about the effects of zinc supplementation on hepatic encephalopathy. The aim of this meta-analysis was to assess the effects of oral zinc supplementation in the treatment of hepatic encephalopathy.

## Methods

### Types of studies

Prospective randomized clinical trials that compared the effects of zinc supplementation with those of no intervention, placebo, or standard therapy on hepatic encephalopathy in patients with liver cirrhosis were included. Trials were included irrespective of publication status, year of publication, or language.

### Types of participants

All adult patients diagnosed with liver cirrhosis using a combination of biochemical and clinical data, regardless of the etiology and treatment, diagnosed with hyperammonemia and hepatic encephalopathy were included.

### Types of interventions

Studies that compared oral zinc supplementation with no intervention, placebo, or other interventions for the management of hepatic encephalopathy were included.

### Types of outcome measures

The primary outcome measures were all-cause mortality, disease-specific mortality (mortality secondary to complications of liver cirrhosis), and severity of encephalopathy as assessed by performance on neuropsychometric tests or recurrence.

The secondary outcome measures were adverse events (all types of adverse events) and quality of life score (measured by any scale).

### Search methods for identification of studies

#### Electronic searches

Relevant randomized trials were identified by searching in CENTRAL, MEDLINE, and EMBASE.

#### Searching other resources

The references in all identified studies were inspected to identify other trials. The first or corresponding author of each included trial, as well as active researchers in the field were contacted for information about unpublished trials and additional information from their own trials.

### Selection of studies

Two authors independently inspected each identified reference and applied the inclusion criteria. For potentially relevant articles or in cases of disagreement between the two reviewers, the full-text article was obtained and inspected independently; if the disagreement could not be solved, a third author reviewed the article. Justification for study exclusion was documented.

### Data extraction and management

Two authors independently extracted the data from the included trials. In cases of disagreement, a third author extracted the data. Extracted data were discussed and this discussion was documented; when necessary, the authors of the original studies were contacted. Justification for study exclusion was documented. Trials were identified by the last name of the first author and the year of publication.

### Assessment of risk of bias in included studies

Two authors independently assessed risk of bias in the trials without masking the trial names. Assessment was conducted according to the Cochrane Handbook for Systematic Reviews of Interventions [13].

### Measures of treatment effect and data analysis

RevMan Analyses software was used for the statistical analysis [14]. Dichotomous data were synthesized and analyzed by calculating the RR and 95% CI for each trial. Continuous data were synthesized and analyzed by calculating the standard mean difference (SMD) between groups for each trial and its 95% CI.

### Assessment of heterogeneity

We checked the heterogeneity of effects across trials by visual inspection of the forest plots and *χ*^2^ and I^2^ tests for heterogeneity. Statistical heterogeneity was defined as *P* > 0.10 (*χ*^2^) or I^2^ > 25%. Whenever heterogeneity was detected, subgroup analysis was performed to assess the effect of potential sources of heterogeneity on the main results.

### Assessment of reporting biases

A funnel plot estimating the precision of trials (plot of logarithm of the RR against the sample size) was used to evaluate asymmetry and detect potential publication bias. In addition, Egger´s test was used to quantify the bias captured by the funnel plot [15].

### Sensitivity analysis

We analyzed the data using both fixed and random-effect models. When both models produced similar estimates, the fixed-effect result was reported; otherwise, we reported the results from both analyses (Additional file 1: Figure S1). Outcomes were analyzed as reported in the trial, either per protocol or as an intention-to-treat.

## Results

### Study selection

**Figure 1:**
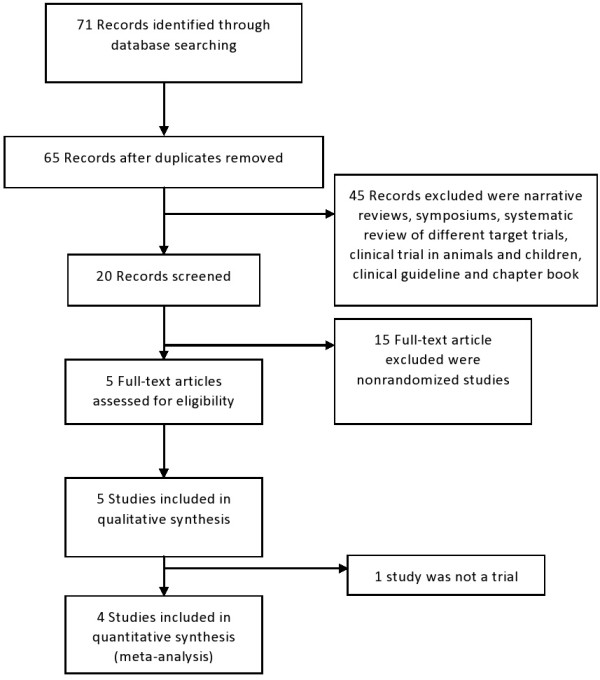
Flow diagram of trial selection.

A total of 65 potential references were retrieved: 36 were narrative reviews, 15 were nonrandomized studies, four were symposium reviews, one was a systematic review of different target trials, one was a clinical trial in animals, one was a trial in children, one was a clinical guideline, and one was a book chapter. Finally, five randomized controlled trials were included in the first analysis, but one study was excluded after a second evaluation of the inclusion criteria (Figure 1).

### Study characteristics

We included four randomized controlled trials designed to evaluate oral zinc supplementation in the treatment of hepatic encephalopathy. The number of patients who received oral zinc supplementation ranged from 20 to 90. A total of 233 patients from three countries, Belgium [10], Italy [11], and Japan [1,16], were included. All studies involved patients with cirrhosis and different stages of encephalopathy. The doses used were zinc sulfate 600 mg/d [16], zinc acetate 600 mg/d [10,11], or polaprezinc 225 mg/d [1] (containing 51 mg of zinc and 174 mg of l-carnosine). All studies were randomized, double-blind, placebo-controlled trials (Table 1).

**Table 1:** Characteristics of trials included in this systematic review and meta-analysis

**Characteristic**	**Reding**[10]	**Bresci**[11]	**Hayashi**[16]	**Takuma**[1]
Place	Brussels, Belgium	Pisa, Italy	Osaka, Japan	Okayama, Japan
Study	Randomized, double-blind, placebo-controlled trial	Randomized, double-blind, placebo-controlled trial	Randomized double-blind, placebo-controlled trial	Randomized, not blinded, placebo-controlled trial
Patients (*n*)	22	90	45	79
Sex (male/female)	15/7	56/34	23/17	40/39
Age (y) (placebo/zinc)*	52.7 ± 13.4/52.1 ± 9.9	49 ± 9/51 ± 9	65.1 ± 11.3/66.0 ± 9.9	66.5 ± 7.4/66.5 ± 5.7
Cause of cirrhosis (viral/alcoholic/other)	ND	50/30/10	38/0/2	58/13/8
Child–Pugh classification (A/B/C)	2/17/3	0/65/25	ND ^1^	15/49/15
Hepatic encephalopathy grade (1/2)	22/0	90/0	ND ^1^	49/30
Baseline serum zinc levels (μg/dL) [placebo/zinc]*	60.3 ± 17.9/64.5 ± 21	52 ± 5/56 ± 6	60.2 ± 9/58.4 ± 9.2	51.6 ± 13.3/48.9 ± 9.3
Baseline NCT (placebo/zinc)	56.2 ± 25.4/55.5 ± 18.9	62 ± 10/66 ± 9	ND	A: 72.6 ± 30.5/78.8 ± 27 B: 141.6 ± 31.3/145.8 ± 30.4
Intervention	Zinc acetate 600 mg/d for 7 d	Zinc acetate 600 mg/d for 6 mo	Zinc sulfate 600 mg/d (blood zinc level <7.6 mol/L) and 200 mg/d (blood zinc level 7.6–10.37 mol/L)	Polaprezinc 225 mg (containing 51 mg of zinc and 174 mg of l-carnosine)/d for 6 mo
Comparison	Placebo	Standard therapy	Branched-chain amino acids	Standard therapy
Outcome reported	Effect of oral zinc on HE	Effect of oral zinc on HE	Effect of oral zinc in patients with hepatic cirrhosis	Effect of oral zinc on HRQOL and HE in patients with liver cirrhosis

*ND* No data, *NCT* Number connection test, *HRQOL* Health-related quality of life, *HE* Hepatic encephalopathy. *mean ± SD.

### Risk of bias within studies

The risk of bias was unclear in all trials. Lack of information precluded a proper evaluation of the risk of bias for all studies.

### Synthesis of results

**Figure 2:**
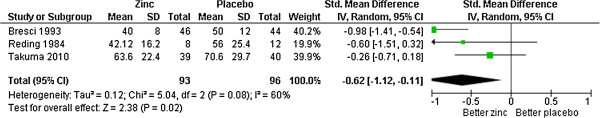
Forest plot for the number connection test results.

**Figure 3:**
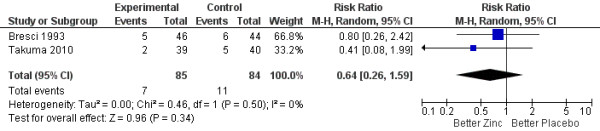
Forest plot for recurrence of hepatic encephalopathy.

Given the large heterogeneity of outcomes across studies, the meta-analysis was restricted to two primary outcomes: number connection test performance and rate of encephalopathy recurrence. Patients treated with oral zinc supplementation experienced a significant improvement in the number connection test performance (SMD –0.62; 95% CI –1.12 to –0.11) compared with patients in the placebo or no supplementation groups (Figure 2). Some heterogeneity of effects (*I*^2^ = 50%) was observed, and stratified analyses were conducted by year of the study and sample size, but no change in the direction or significance of the effect was observed (data not shown). The funnel plot shows no evidence of publication bias (Additional file 2: Figure S2). No reduction was observed in the encephalopathy recurrence rate (RR 0.64; 95% CI 0.26 to 1.59) (Figure 3).

Reding et al. [10] studied the use of oral zinc supplementation in a double-blind randomized trial involving 22 patients with chronic encephalopathy. The zinc group received zinc acetate 600 mg/d. Compared to placebo, the zinc group showed improved performance in the number connection test (56 ± 25.4 and 42.12 ± 16.2 seconds, respectively).

Bresci et al. [11] assessed the effect of long-term zinc supplementation in 90 patients with cirrhosis with stable recurrent hepatic encephalopathy. Oral zinc supplementation (zinc acetate 600 mg/d) in addition to standard therapy normalized the serum zinc levels. Performance in the number connection test (40 ± 8 vs. 50 ±12 seconds), as well as in the portal systemic encephalopathy index improved in the treated compared to placebo group (0.15 vs. 0.19). The treated group experienced less recurrence of encephalopathy; after six months 88.6% of patients in the treated group had no detectable signs of hepatic encephalopathy, compared to 86% in the placebo group.

Hayashi et al. [16] reported improved nitrogen metabolism in patients with liver cirrhosis after administration of branched-chain amino acids and zinc. Forty patients with liver cirrhosis, low serum albumin, and low zinc levels were randomized to receive either branched-chain amino acids alone or a combination of branched-chain amino acids and zinc supplements. Blood ammonia levels tended to increase in the amino acid group, while it decreased in the supplemented zinc group (post/pre change ratio of blood ammonia 1.22 ± 0.38 and 0.87 ± 0.26, P = 0.003, respectively). The Fischer ratio increased in both groups, but showed a sharper increase in the zinc-supplemented group (post/pre change ratio 1.45 ± 0.48 and 1.67 ± 0.5, P = 0.093, respectively).

Takuma et al. [1] found that zinc supplementation was effective in treating hepatic encephalopathy and improving health-related quality of life (particular physical functioning, role-physical, and physical component scale). Seventy-nine patients with cirrhosis and hepatic encephalopathy were randomized to receive 225 mg of polaprezinc in addition to standard therapies. After six months zinc supplementation improved the Physical Component Scale score (P = 0.04) and the Child–Pugh score (7.8 ± 1.6 vs. 7.2 ± 1.4, P = 0.04), and significantly decreased hepatic encephalopathy grade (1.3 ± 0.9 vs. 0.9 ± 0.9, P = 0.03) and blood ammonia levels (112.0 μg/dL ± 56.3 vs. 90.4 μg/dL ± 33.4, P = 0.01). In this study one patient discontinued the treatment due to an adverse event (nausea and vomiting).

## Discussion

In this meta-analysis, we included four randomized controlled trials evaluating the effect of oral zinc supplementation over hepatic encephalopathy. Three studies reported data on number connection test; all three showed an improvement in performance in the zinc group compared to placebo or standard therapy. This improvement suggests a beneficial effect of oral zinc in encephalopathy patients. Two studies reported data on encephalopathy recurrence rate. Both studies observed lower recurrence rates in the zinc groups, suggesting a beneficial effect of zinc; however, given the small sample size, confidence intervals were wide and failed to reach statistical significance.

Hepatic encephalopathy is characterized at the neurophysiological level by disturbed corticocortical and corticomuscular coupling, and at the cellular level by primary gliopathy [2,5,17,18]. Ammonia is a key pathophysiological factor in hepatic encephalopathy [18,19]. In the brain, ammonia is detoxified by astrocytes through a reaction catalyzed by glutamine synthetase; an increased brain glutamine/glutamate ratio is associated with decreased myoinositol, reflecting compensation for glial edema [20-23]. Swollen astrocytes predispose to neuronal dysfunction by impairing their regulatory activity against the increase in protein tyrosine nitration and the formation of reactive oxygen and nitrogen oxide species including nitric oxide. If not counteracted, these reactions promote RNA oxidation, which prompts gene expression and the transcription of altered proteins [2,5,6,18,19,21,24].

Cytokines or lipopolysaccharides could induce the formation of nitrogen oxide species and trigger zinc release from metallothioneins, the principal zinc storage protein. A fluctuation in intracellular zinc levels modulates signal transduction, transcription factor activity, and gene expression, causing hepatic encephalopathy symptoms. Zinc deficiency is associated with disturbances in learning, memory, and emotional stability and is accompanied by hyperammonemia. Zinc supplementation has shown to reduce ammonia levels in experimental animals and humans through hepatic urea synthesis stimulation and glutamine synthesis in skeletal muscle [2,6-8,12,18,19,21,25].

The present meta-analysis is limited by the small number and poor quality of trials included. Available trials studied heterogeneous outcomes and failed to measure critical outcomes such as quality of life. This hinders the ability to draw conclusions about the value of oral zinc supplementation in the treatment of hepatic encephalopathy. Additionally, little information regarding the clinical importance of the different zinc formulations used in the trials was available.

In conclusion, oral zinc supplementation improved performance on the number connection test, but there is no clear evidence that supplementation improves encephalopathy or encephalopathy-related quality of life. More trials are needed to evaluate the use of oral zinc supplementation in patients with liver cirrhosis and hepatic encephalopathy.

## Competing interests

The authors declare that no competing interests exist.

## Authors’ contributions

NCC-T: protocol writing, searching, trial selection, data extraction, report writing, drafting the article, and final approval of the manuscript. AC-A: protocol writing, searching, trial selection, data extraction, report writing, drafting the article, and final approval of the manuscript. TB-G: report writing, drafting the article, and final approval of the manuscript. NM-S: report writing, drafting the article, and final approval of the manuscript. MU\: report writing, drafting the article, and final approval of the manuscript. All authors read and approved the final manuscript.

## Supplementary Material

Additional file 1 Fixed model. Click here for file

Additional file 2 Funnel plot. Click here for file
