# Supplementary figures and images for: A systematic review and meta-analysis of the use of oral zinc in the treatment of hepatic encephalopathy
Source: Nutr J. 2013 Jun 6;12:74. doi: 10.1186/1475-2891-12-74 (PMC3689058; doi:10.1186/1475-2891-12-74)

## Slide 1
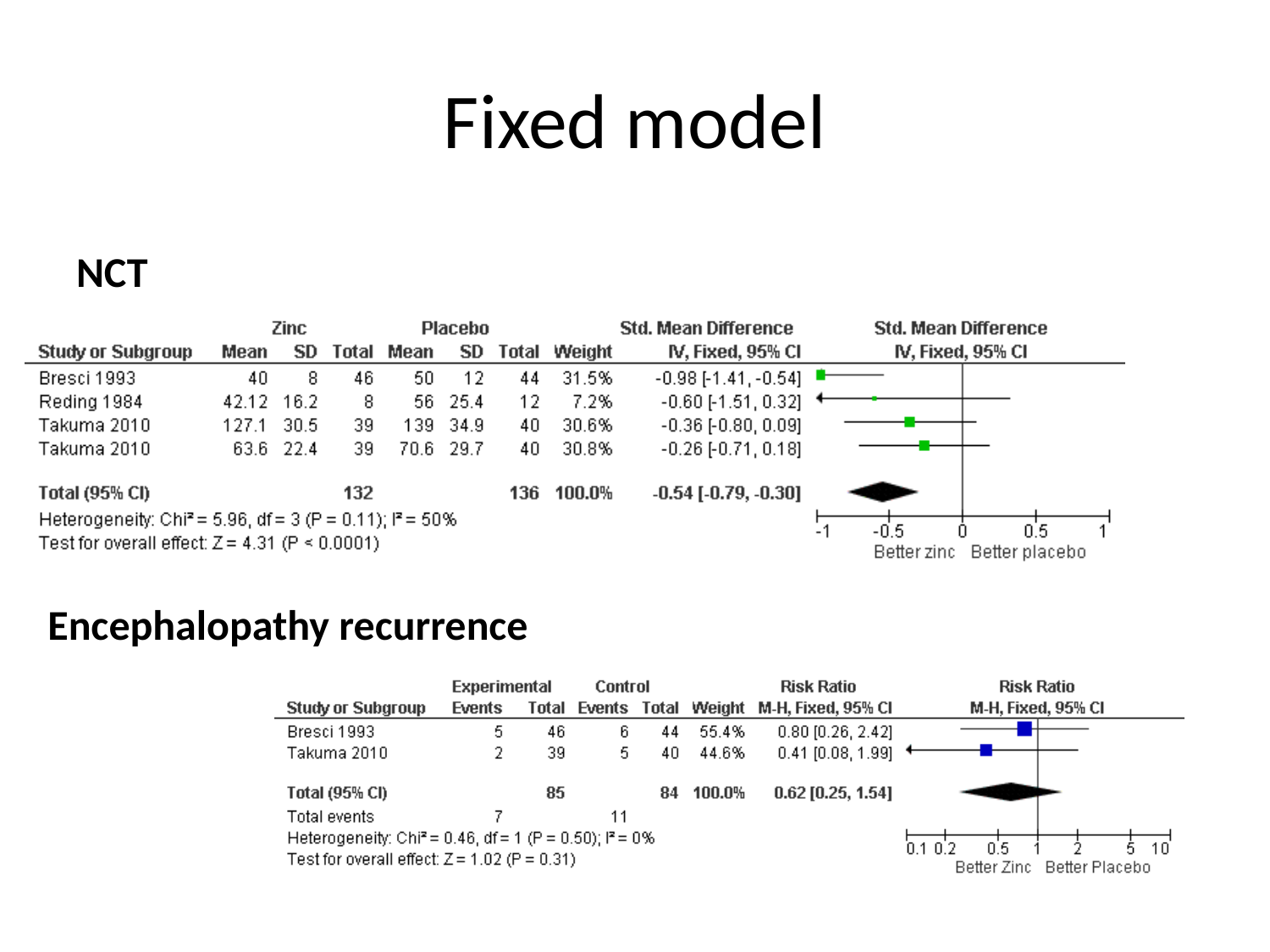

# Fixed model
NCT
Encephalopathy recurrence

Supplement: Additional file 1 — Fixed model. [file 1475-2891-12-74-S1.pptx]

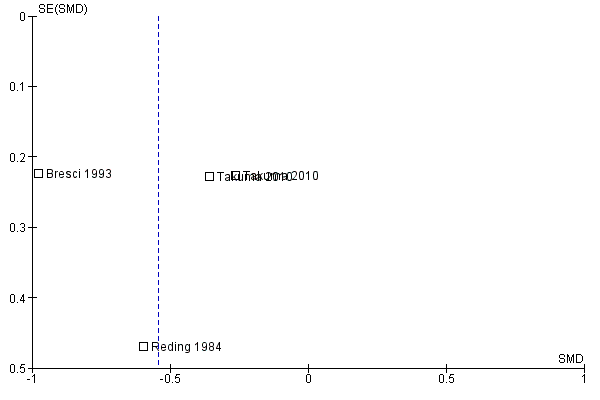

Supplement: Additional file 2 — Funnel plot. [file 1475-2891-12-74-S2.png]
